# Coastal Microbial Communities Disrupted During the 2018 Hurricane Season in Outer Banks, North Carolina

**DOI:** 10.3389/fmicb.2022.816573

**Published:** 2022-06-09

**Authors:** Cody E. Garrison, Sara Roozbehi, Siddhartha Mitra, D. Reide Corbett, Erin K. Field

**Affiliations:** ^1^Department of Biology, East Carolina University, Greenville, NC, United States; ^2^Department of Geological Sciences, East Carolina University, Greenville, NC, United States; ^3^Integrated Coastal Programs, East Carolina University, Greenville, NC, United States

**Keywords:** microbial communities, coastal ecosystems, metagenomics, hurricanes, global change

## Abstract

Hurricane frequencies and intensities are expected to increase under warming climate scenarios, increasing potential to disrupt microbial communities from steady-state conditions and alter ecosystem function. This study shows the impact of hurricane season on microbial community dynamics within the barrier island system of Outer Banks, North Carolina. We found that the passage of two sequential energetic hurricanes in 2018 (Florence and Michael) were correlated with shifts in total and active (DNA and RNA) portions of bacterial communities but not in archaeal communities, and within surface waters but not within the sediment. These microbial community shifts were distinct from non-hurricane season conditions, suggesting significant implications for nutrient cycling in nearshore and offshore environments. Hurricane-influenced marine sites in the coastal North Atlantic region had lower microbial community evenness and Shannon diversity, in addition to increased relative abundance of copiotrophic microbes compared to non-hurricane conditions. The abundance of functional genes associated with carbon and nitrogen cycling pathways were also correlated with the storm season, potentially shifting microbial communities at offshore sites from autotroph-dominated to heterotroph-dominated and leading to impacts on local carbon budgets. Understanding the geographic- and system-dependent responses of coastal microbial communities to extreme storm disturbances is critical for predicting impacts to nutrient cycling and ecosystem stability in current and future climate scenarios.

## Introduction

An increasingly warmer global climate has been putatively linked to higher intensity and more frequent extreme storm events (i.e., tropical storms, hurricanes, and typhoons; Emanuel, 2005; Groisman et al., 2005; Knutson et al., 2010; Kunkel et al., 2010; Holland and Bruyère, 2014). The extent that storm events might reshape local aquatic microbial communities remains unclear. Historical data suggest that the coast of North Carolina is at particularly high risk for tropical cyclones and hurricanes (Kunkel et al., 2010). The inlets associated with the Outer Banks barrier island system in North Carolina (e.g., Oregon Inlet) serve as conduits for freshwater and terrigenous materials from the East Coast of the United States to the boundary of the Gulf Stream at the edge of the continental shelf (~60 km). As a result, disruptions to coastal microbial communities in this area may have a potential to affect nutrient flow (i.e., C, N, and P) within the coastal North Atlantic region and into the Gulf Stream. Offshore marine sites closer to the Gulf Stream have important supporting roles as net sinks for atmospheric CO_2_ due to their high primary production, which helps to balance increasing amounts of net sources linked to climate change (Biscaye et al., 1994; IPCC, 2007; Chen and Borges, 2009). Extreme storm events can lead to erosion and runoff of limited nutrients, followed by increased microbial production and respiration (Bauer et al., 2013; Bianchi et al., 2013; Hounshell et al., 2019: Yan et al., 2020). In such situations, coastal and offshore aquatic environments may temporarily switch from net autotrophic to net heterotrophic. This would have important implications for regional carbon budgets with increased atmospheric CO_2_ inputs being counter-productive for warming global climates.

**Figure 1:**
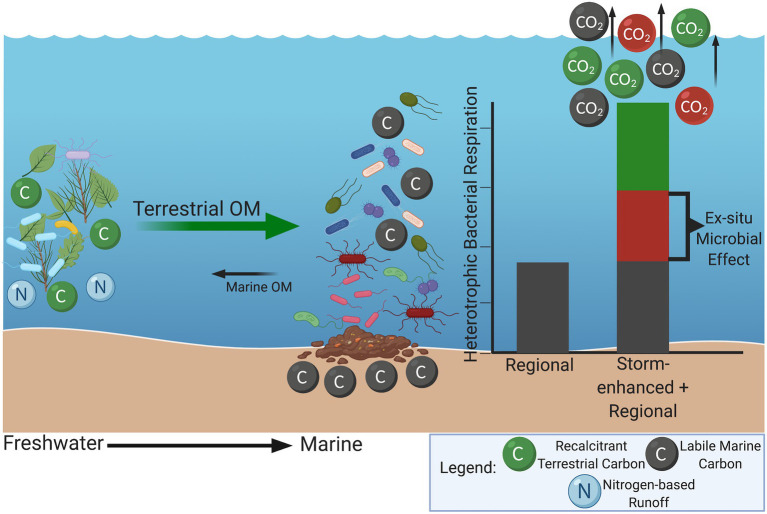
Depiction of storm-enhanced microbial production at the interface of terrestrial and marine systems. Input of recalcitrant, high-molecular-weight plant material, terrestrial microbes, and nitrogen-based runoff into labile marine systems increases microbial biomass and production of extracellular enzymes allowing enhanced breakdown of recalcitrant material. This additive effect of higher bacterial production and respiration can be referred to as the “*ex-situ* microbial effect” as seen in red on the bar graph. Green portion of the bar graph represents contribution from terrestrial heterotrophic respiration. Created with BioRender.com.

Extreme storm events are often associated with rapid input of allochthonous nutrients leading to significant microbial community disruptions (Bergström and Jansson, 2000; Zhang et al., 2019). Mechanisms of community disruptions range from eutrophication and hypoxia to introduction of herbicides, dangerous chemicals, and pathogenic bacteria from runoff (Solo-Gabriele et al., 2000; Anderson and Taylor, 2001; Chen et al., 2012, 2018; Lewis et al., 2012; Mistri et al., 2019). Storm events and associated floodwater surging can also lead to increased input of recalcitrant, high-molecular-weight plant material (e.g., lignin) and terrestrial microbes into marine systems leading to anomalous periods of increased microbial production and respiration (Bingeman et al., 1953; Kuzyakov et al., 2000; Bianchi, 2011). This “storm-enhanced microbial production” is driven by exposure of these terrestrial microbes to the labile organic material inherently found in marine systems, in combination with highly reactive nitrogen-based runoff from inland agricultural areas and wetlands (Figure 1). The subsequent microbial feeding frenzy increases microbial biomass and production of extracellular enzymes allowing for more efficient breakdown of the freshly introduced recalcitrant terrestrial material (Bianchi, 2011). This transboundary, or “*ex-situ* microbial effect,” increases microbial production and respiration beyond that which would occur separately within terrestrial and marine systems without storm influence (Wisnoski et al., 2020). Terrestrial, or freshwater environments have inherently different microbial community compositions compared to their marine counterparts, and this *ex-situ* microbial effect could produce a more mixed community composition rather than the typical gradient observed at these environmental interfaces (Methé et al., 1998; Logares et al., 2009).

Short-term environmental disturbances, or pulse disturbances (e.g., storm events), can have serious consequences for microbial community function depending on the resistance and resilience of the community (Shade et al., 2012). Resistance can be defined as the ability to endure perturbation caused by a disturbance, while resilience can be defined as the ability to recover from perturbation (Connell and Ghedini, 2015; Nimmo et al., 2015). Higher resistance represents a lower sensitivity to disturbance, while higher resilience results in faster community recovery following the event. Impacts from storm disturbance events are also expected to be geographically context dependent. For example, storm impacts could be controlled by proximity to urban or agricultural environments with potential for runoff, local topography and hydrology of the coastal region, and characteristics of the storm (e.g., intensity, duration, and path). Previous studies from different locations have shown variable degrees of impact from extreme storms toward microbial communities (Okinawa, Japan: Ares et al., 2020; Pamlico Sound, North Carolina: Paerl et al., 2001; Peierls et al., 2003; Oahu, Hawaii: De Carlo et al., 2007; Yeo et al., 2013; New Orleans, Louisiana: Sinigalliano et al., 2007; and Houston, Texas: Steichen et al., 2020). Increased understanding of geographic region-specific shifts in microbial community composition and functional potential following extreme storms is vital to interpreting disruptions to nutrient cycling processes and the overall health of a coastal ecosystem.

The coastal North Carolina region in particular faces increased risk of storm-induced microbial community disruption due to long residence times (~1 year) characterizing the local Albemarle-Pamlico estuarine system that flows into coastal North Carolina marine waters (Paerl et al., 2001). This region has seen doubling of annual nitrogen loads, tripling of annual phosphorus loads, and enough organic carbon input to change the coastal region from a net sink to a net source of CO_2_ during years with at least one major storm (~10^6^ kg N, ~10^5^ kg P, and ~ 10^7^ kg DOC input over a 2-week period following Florence in 2018; Paerl et al., 2018, 2020). Hurricane Matthew in 2016 significantly impacted the local ecosystem, accounting for ~25% of the total annual carbon input to the Neuse-Pamlico Estuary (Osburn et al., 2019). Eastern North Carolina also exhibits high concentrations of agricultural and animal production that have serious coastal ecosystem consequences as a result of storm-induced floodwaters and runoff (Mallin and Cahoon, 2003; Casteel et al., 2006). Increased relative abundance of foodborne zoonotic pathogens due to Hurricane Florence has been reported for watersheds of North Carolina (Niedermeyer et al., 2020). It is important to understand the impact of environmental fluctuations on coastal North Carolina microbial communities and its subsequent influence on steady-state conditions, resistance and resiliency, and future climate change scenarios for this ecosystem.

This study aims to answer the question “did the 2018 hurricane season in NC disrupt microbial community and ecosystem functional potential?” To that end, we analyzed the coastal microbial communities during the 2018 hurricane season and compared them to similar samples taken during non-hurricane-season months in 2016 and 2019. We hypothesized that coastal North Carolina exhibited shifts in microbial community composition, putative activity, and functional potential during the 2018 hurricane season. Two separate hurricanes made landfall on coastal North Carolina in late 2018: Hurricane Florence on 14 September 2018 and Hurricane Michael on 11 October 2018. Florence approached from the Southeast Atlantic, made landfall near Wilmington, NC, and brought record-breaking rainfall >76 cm over 7 days and wind gusts >45 m s^−1^ (National Weather Service, 2018; Supplementary Figure S1). Florence was a slow-moving and persistent storm system that created flood levels for inland rivers that have rarely been seen before. Floodwaters in some Outer Banks areas reached 120 cm above sea level (National Weather Service, 2018). Meanwhile, floodwaters across some inland rivers did not start to recede for nearly 3 weeks after Florence made landfall (USGS, 2018). This delayed recession is expected to cause continued impacts to the coastal marine ecosystem for weeks following the storm. Hurricane Michael further added to the ecosystem disruption, with wind gusts over 33 m s^−1^ and floodwaters rising again, this time reaching 94 cm above sea level (National Weather Service, 2018). Michael approached from the Gulf of Mexico and made landfall on the Florida Panhandle before moving across Southeast United States. The combination of two extreme storm events relatively close together in timeframe provide a unique view of sequential-storm conditions that may be more frequently observed under warmer future conditions if the link between warming climate and increased frequency of storm events holds true. The analysis of both DNA and RNA from sediment and surface water microbial communities following both storm events and throughout the latter portion of the 2018 hurricane season provides insight into the impact to microbial community function and nutrient cycling. Microbial RNA surveys can identify “active” members of microbial communities at the time of sampling while “total” microbial community measurements can be obtained from DNA-based community profiles (Campbell et al., 2009). To our knowledge, RNA-based microbial profiling has not been used previously in extreme storm studies on microbial communities and could provide a benchmark for correlating DNA surveys to active functional members of hurricane-influenced communities affecting ecosystem nutrient flow. Analysis of functional gene abundances *via* shotgun metagenomic sequencing further links putative biological function to microbial taxonomy associated with extreme storm impacts on microbial communities. These combined methods ultimately provide geographic region-specific insight into extreme storm influence on microbial community functional potential and their implications for nutrient cycling across the ecosystem. These results will be critical for understanding the future impacts of climate-induced shifts in frequency and severity of extreme storm events affecting all domains of life.

## Materials and Methods

### Sample Collections and Processing

**Figure 2:**
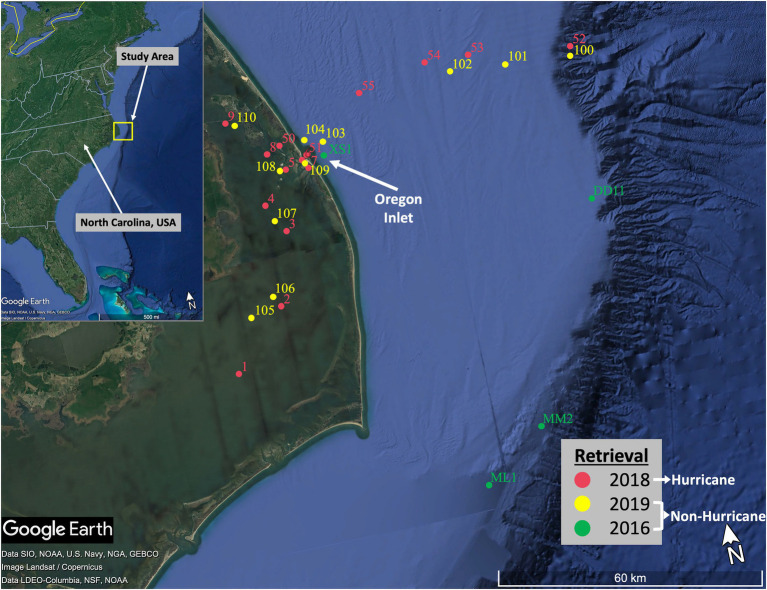
Map of sampling sites from each retrieval. Sites represent both the northern (i.e., the Albemarle Sound) and southern region (i.e., the Pamlico Sound) of the Albemarle-Pamlico estuary, as well as within Oregon Inlet and along an E-W transect toward the edge of the continental shelf and Gulf Stream.

Fifteen surface water samples and thirteen sediment samples were collected within the coastal NC system following Hurricane Florence and Michael. Sampling was conducted as quickly as was feasible due to equipment issues, weather patterns, and accessibility of sites. Samples were collected on 25 October, 26 October, and 30 November 2018 from the Coastal Studies Institute research vessel R/V *Blackbeard*, in waters with salinities ranging from 4.2 within the estuary to 33.5 closest to the Gulf Stream (Figure 2; Supplementary Table S1). The slow recession of inland floodwaters (~3 weeks after Florence made landfall; USGS, 2018) combined with the long residence times characterizing the coastal estuarine system (Paerl et al., 2001) gave us confidence that we were still able to capture the effects of the storms on the ecosystem. These “hurricane-influenced” samples were then compared to non-hurricane season samples collected in Summer 2019 using the same sample collection methods. Extreme storm events inherently have the potential for extreme changes to microbial communities and ecosystem function, yet they are severely understudied because of the associated difficulty in timing and general safety of sample collection. We realize that seasonal variabilities have the potential to impact our results; however, the unpredictability of trying to sample again during the same season on the following year (i.e., Fall 2019) could present new unexpected hurricanes at any moment that would compromise such a sampling plan. It was determined that the best representation of “baseline” conditions would be Summer 2019, while also being cognizant of potential seasonal influences during analysis of the results. These non-hurricane-influenced surface water and sediment samples were collected on 2 July and 3 July 2019 (11 surface water and 12 sediment; salinities ranging 8.5–29.9). Sites for the 2018 and 2019 sampling efforts were chosen with the intention of surveying microbial communities exposed to both the northern (i.e., the Albemarle Sound) and the southern regions (i.e., the Pamlico Sound) of the Albemarle-Pamlico estuary, as well as within Oregon Inlet and along an E-W transect toward the edge of the continental shelf and Gulf Stream. While additional samples would have been preferable from multiple hurricane seasons and across multiple years, the scope of the project was focused on 1 year as a first step toward investigating potential impacts due to hurricane disturbances. In addition to the samples above, we were able to process preserved surface water and sediment samples taken in March 2016 from a separate cruise onboard the R/V *Neil Armstrong*. These 2016 samples served as a fortuitous supplement to the data analysis in this study and to provide additional evidence that samples taken during 2018 represent a disrupted coastal microbial community.

Surface water samples were collected by filling sterilized polycarbonate plastic containers with surface water from the side of the boat. Bulk sediment samples were collected using a Van Veen grab sampler wherever feasible based on water depth and strength of current, and subsamples for analysis were collected aseptically from bulk samples avoiding edge effects. Surface water from each site was stored on ice immediately following collection and during transport to the lab. All water samples were aseptically filtered onto MilliporeSigma™ polyethersulfone 0.22 μm filters to capture microbial cells. Filtered surface water volumes ranged from 200 ml to 4 L due to some water samples having higher turbidity than others, which led to filters being clogged and/or saturated with particulate material at different rates. Nonetheless, all analyses presented in this study utilize relative microbial abundance rather than absolute abundance, and thus highlight trends in the proportion of taxa rather than changes in the total number of cells detected. We recognize that previous studies have suggested the potential for distortion of planktonic microbial community diversity estimates when comparing different volumes of water filtered (Padilla et al., 2015). However, that study used “pre-filtered” samples (through 1.6 μm pore size) to illustrate biases in studies on particle-associated vs. free-living. We did not perform pre-filtering as we are interested in both particle-associated and free-living communities. Our samples were taken from different sampling sites with different turbidities, which made it impossible to filter samples using all the same volumes. All things considered, variations in filtered surface water volumes in this study should not affect the results reported.

Whole, separate filters were used for individual nucleic acid extraction methods (DNA and RNA) in order to maximize yields from the same well-mixed collection. RNA sampling improves overall analyses because total microbial communities identified from DNA profiles may also include extracellular DNA (Dell’Anno et al., 1998), as well as dead or dormant microbial cells that are likely not contributing to nutrient cycling or ecosystem functioning (Freedman et al., 2015; Cardoso et al., 2017). Note that some limitations exist for using DNA and RNA extractions to infer microbial community functional potential, as relative abundance of nucleic acids does not always correlate with activity nor total number of cells (Blazewicz et al., 2013). Surface water samples were filtered within 24 h of collection to prevent changes in microbial community composition and RNA expression. Filters containing microbial cells were stored at −80°C until DNA and RNA extraction using MoBio DNeasy and RNeasy Power Water kits (Qiagen, Inc.). Sediment samples (~0.25 g) were extracted for DNA and RNA using MoBio DNeasy and RNeasy Power Soil kits (Qiagen, Inc.). Both DNA and RNA were extracted separately to identify the “total” and “active” microbial communities within surface waters and sediment, respectively, at the time of sampling. Extracted RNA was converted to cDNA using a High-capacity RNA-to-cDNA reverse transcription kit (Thermo Fisher Applied Biosystems) to enable amplicon sequencing of the “active” portion of the microbial community.

Water quality measurements were taken for 2018 and 2019 surface water samples and included total suspended solids (TSS), dissolved organic carbon (DOC), and total dissolved nitrogen (TDN). Water samples were filtered through combusted (450°C for 4 h) and pre-weighed 0.7 μm pore size glass fiber filters (GFF) to isolate suspended sediments. Filters were then air-dried at room temperature until there was no further change in dry weight. Total suspended sediment was calculated using the Equation 1, below:

$\mathrm{TSS}=\left[\begin{array}{l} \left(\mathrm{filter}+\mathrm{sediment}\;\mathrm{dry}\;\mathrm{weight}\right)\\ -\mathrm{filter tare weight} \end{array}\right]/\mathrm{volume of water filtered}$

The filtrate samples were acidified with 2 N HCl and analyzed for DOC and TDN using a Shimadzu TOC-VCPN/TNM-1 analyzer *via* the combustion catalytic oxidation method (flow rate = 150 ml/min; 680–720 WC) and chemiluminescence detector. Note that TSS, DOC, and TDN were isolated in the filtrate from water samples filtered at 0.7 μm while microbial community techniques in this study were associated with the 0.2 μm retentate.

### Nucleic Acid Sequencing and Analysis of Results

Extracted DNA and cDNA were used to generate 16S rRNA gene amplicon libraries for sequencing to identify microbial community compositions. 16S rRNA gene amplicon sequencing was performed by CGEB Integrated Microbiome Resource (Dalhousie University, Halifax, Canada). Amplicon sequencing utilized a Phusion polymerase PCR amplification method (Comeau et al., 2017) on an Illumina MiSeq platform, targeting the V4-V5 region of the 16S rRNA gene to capture the largest majority of microbial taxonomy (Yang et al., 2016). Amplicon sequence data were processed using mothur v.1.41.3 (Schloss et al., 2009) and its associated analysis pipeline (Kozich et al., 2013). Taxonomy was determined based on 97% OTU classifications. Canonical Correspondence Analysis plots were created in R using vegan and ggplot2 packages with the metaMDS function and a Bray–Curtis similarity matrix (R Core Team, 2013). An analysis of similarities (ANOSIM; Clarke, 1993; Warton et al., 2012) was used to determine significant differences between sample groups in R, and a permutational multivariate analysis of variance (PERMANOVA; Anderson, 2017; Adonis test in R) was used to determine significance and correlation between environmental variables and microbial community composition. The vegan package in R was further used to determine community diversity indices (richness, evenness, and Shannon diversity; Shannon, 1948) across all samples. Boxplots depict five key summary statistics including the median, 25% and 75% percentiles, and the feasible range of the data (±1.5 × the interquartile range) represented by the two whiskers. Significant differences between sample groups’ diversity indices were confirmed using a Mann–Whitney U test (McKnight and Najab, 2010; wilcox.test function in R). A similarity percentage analysis (SIMPER; Clarke, 1993; Warton et al., 2012) was used to evaluate microbial taxa with the most significant influence toward differences in community composition between sample groups. rRNA (*rrn*) copy numbers were determined for each sample using RDP classifier v.2.13 (Wang et al., 2007) combined with the *rrn* operon database (Stoddard et al., 2015).^1^

Ten DNA samples were chosen for shotgun metagenomic sequencing with the intention of providing the most comprehensive evaluation of sample types, sample sites, and sampling years. Each sample was analyzed for changes in functional gene potential across hurricane-influenced and non-hurricane-influenced conditions (hurricane-influenced: 2018: one marine and two estuarine surface water samples, one sediment sample; non-hurricane-influenced: 2016: two marine surface water samples; 2019: two marine and one estuarine surface water samples, one sediment sample) These samples were sequenced using shotgun metagenomics by CGEB Integrated Microbiome Resource (Dalhousie University, Halifax, Canada). An Illumina Nextera Flexkit method (Comeau et al., 2017) was used with an Illumina NextSeq platform at 2x sequencing depth in order to obtain roughly 8 million paired-end reads (150 + 150 bp; i.e., 16 million single reads) and roughly 2.4 Gb per sample. Resulting raw reads were then trimmed using TrimGalore v.0.4.5 (Krueger, 2012) and checked for quality using FastQC v.0.11.8 (Andrews, 2010). After trimming, reads were concatenated and assembled using SPAdes v.3.13.0 (Bankevich et al., 2012). MG-RAST v.4.0.3 (Meyer et al., 2008) was used to annotate assembled contigs, and annotations were manually searched for carbon cycling genes (e.g., lignin-degradation genes) according to those described by Janusz et al. (2017). KBase UI v.2.2.1 (Arkin et al., 2018) was used to run DRAM (Shaffer et al., 2020) for metabolic profile functional annotations of other common carbon and nitrogen cycling pathways. Depiction of storm-enhanced microbial production (Figure 1) was created using BioRender©.^2^

## Results

### Disruptions to Total (DNA) and Active (RNA) Bacterial but Not Archaeal Community Compositions During the 2018 Hurricane Season, and Within Surface Waters but Not Within the Sediment

**Figure 3:**
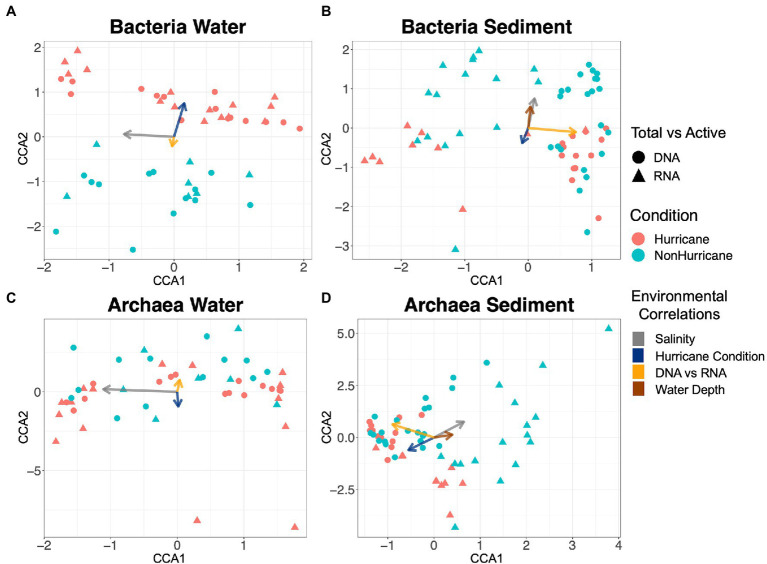
Canonical Correspondence Analysis plots showing similarities in microbial community composition across samples. Each data point is one sample, and distance between points represent degree of similarity in community composition. Arrows represent influence from environmental variables, and length of arrow represents strength of correlation. **(A)** Hurricane conditions led to significant differences within surface water bacterial communities compared to non-hurricane conditions (ANOSIM test: *R* = 0.185, *p* < 0.001), with community composition significantly correlated with salinity and putative cell activity (PERMANOVA test: Salinity: *R*^2^ = 0.357, *p* < 0.05; Hurricane conditions: *R*^2^ = 0.105, *p* < 0.05; DNA vs. RNA: *R^2^* = 0.102, *p* < 0.05); **(B)** Bacterial sediment communities were most strongly correlated with putative cell activity, followed by salinity and water depth (DNA vs. RNA: *R*^2^ = 0.31, *p* < 0.05; Salinity: *R*^2^ = 0.059, *p* < 0.05; Water depth: *R*^2^ = 0.043, *p* < 0.05); **(C)** Archaeal surface water communities were correlated only with salinity (*R*^2^ = 0.377, *p* < 0.05); and **(D)** Archaeal sediment communities were most strongly controlled by putative cell activity, followed by salinity and water depth (DNA vs. RNA: *R*^2^ = 0.305, *p* < 0.05; Salinity: *R*^2^ = 0.152, *p* < 0.05; Water depth: *R*^2^ = 0.060, *p* < 0.05).

Bacterial communities isolated from surface waters following Hurricanes Florence and Michael during the 2018 hurricane season (hurricane conditions) were distinct from bacterial community samples taken in 2016 and 2019 (non-hurricane conditions; ANOSIM test: *R* = 0.185, *p* < 0.001; Figure 3A). Salinity was the most influential variable affecting community composition in surface waters, followed by sampling period (hurricane or non-hurricane conditions) and subsequently by putative cell activity (DNA vs. RNA), although all three were significant (Figure 3A; PERMANOVA test: salinity: *R*^2^ = 0.357, *p* < 0.05; hurricane conditions: *R*^2^ = 0.105, *p* < 0.05; DNA vs. RNA: *R*^2^ = 0.102, *p* < 0.05). Note that the environmental variable indicated by “DNA vs. RNA” represents the microbial composition that results from independent extractions of either DNA or RNA in order to infer differences between the total and active community, respectively. Also note that surface water bacterial communities were correlated with hurricane conditions even without inclusion of the supplemental 2016 samples (ANOSIM test: *R* = 0.146, *p* < 0.05). Further, bacterial communities in non-hurricane condition samples from 2016 and 2019 were more similar to each other than to those taken during the 2018 hurricane season (Supplementary Figure S2), but the limited number of samples from 2016 limit statistical significance. Although samples were taken across multiple days and months during the 2018 hurricane season, it is difficult to assess variability or differences across hurricane season because sampling days were separated based on salinity (i.e., offshore sites were sampled 1 day, while nearshore sites were sampled a different day). Sediment bacterial community compositions were not significantly correlated with the 2018 hurricane season but did exhibit significant differences between putative cell activity, salinity, and water depth (DNA vs. RNA: *R*^2^ = 0.31, *p* < 0.05; salinity: *R*^2^ = 0.059, *p* < 0.05; water depth: *R*^2^ = 0.043, *p* < 0.05; hurricane conditions: *R*^2^ = 0.019, *p* = 0.11; Figure 3B).

Archaeal community analyses have not previously been addressed in studies focused on the impact of storm events on microbial communities, and our results demonstrate a clear contrast in response of archaeal communities to extreme storms compared to bacterial communities. Archaea within surface waters had no significant correlation with hurricane conditions or putative cell activity, while the effect of salinity was significant (salinity: *R*^2^ = 0.377, *p* < 0.05; DNA vs. RNA: *R*^2^ = 0.023, *p* = 0.13; hurricane conditions: *R*^2^ = 0.011, *p* = 0.41; Figure 3C). Archaeal sediment communities also had no significant correlation with hurricane conditions, while putative cell activity, salinity, and water depth were all significant (DNA vs. RNA: *R*^2^ = 0.305, *p* < 0.05; salinity: *R*^2^ = 0.152, *p* < 0.05; water depth: *R*^2^ = 0.060, *p* < 0.05; hurricane conditions: *R*^2^ = 0.019, *p* = 0.07; Figure 3D). When evaluating any potential functional redundancy in microbial communities across environment types, both bacterial and archaeal communities within surface waters exhibited very little similarity to those within the sediment during hurricane conditions (ANOSIM test: *R* = 0.971, *p* < 0.0001).

### Lower Shannon Diversity, Lower Taxonomic Evenness, and Increased Abundance of Copiotrophs at Hurricane-Influenced Marine Sites

**Figure 4:**
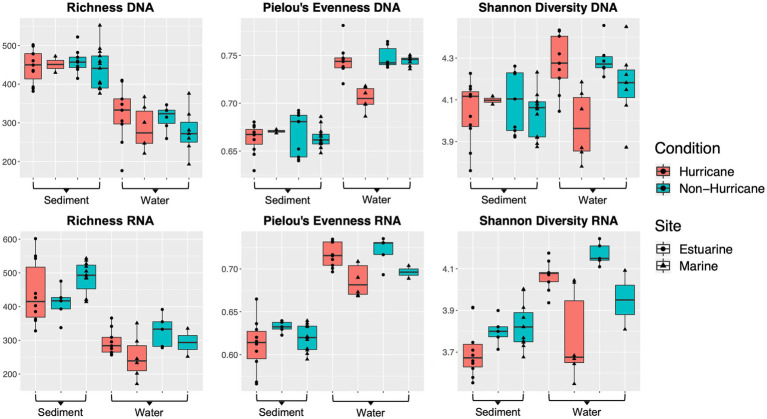
Boxplots of microbial community diversity indices. Microbial communities at marine sites (>25 ppt) exhibited lower Shannon diversity (Mann–Whitney U test: *W* = 7, *p* < 0.01) and lower taxonomic evenness (*W* = 0, *p* < 0.001) within surface waters after hurricane conditions compared to non-hurricane conditions.

**Figure 5:**
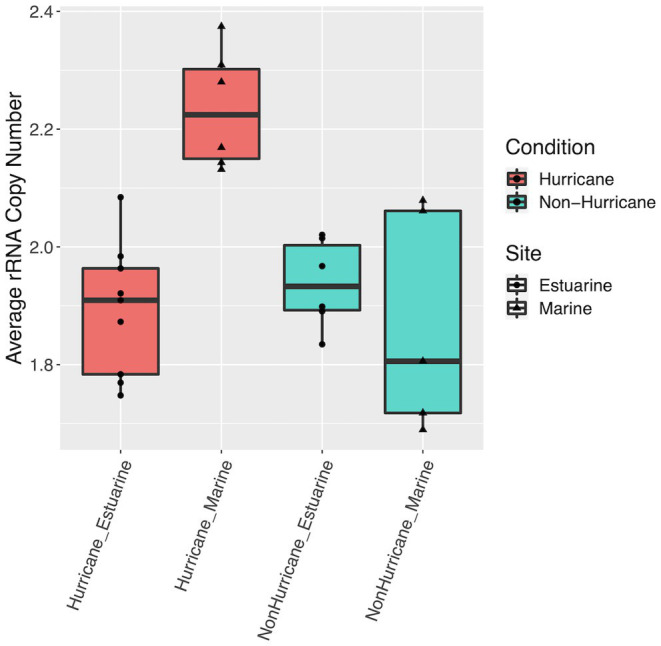
Boxplots of average rRNA (*rrn*) copy number across samples. Hurricane condition marine sites had higher average copy number compared to hurricane condition estuarine sites and all sites from non-hurricane conditions (Mann–Whitney U-test: *W* = 120, *p* < 0.001).

Microbial community DNA from marine sites (>25 ppt) had lower Shannon diversity (Mann–Whitney U test: *W* = 7, *p* < 0.01) and lower taxonomic evenness (*W* = 0, *p* < 0.001) within surface waters during hurricane conditions compared to non-hurricane conditions (Figure 4). Surface waters had no difference in microbial community richness between conditions. There was also no significant difference across all diversity indices within the sediment. RNA-based community indices followed roughly the same trends as DNA-based results above, although statistical significance was limited due to poor RNA yields for several surface water samples from non-hurricane marine sites. The decrease in diversity at marine sites was analyzed further using rRNA gene copy number as a proxy for inferring metabolic lifestyle. Microbes with higher rRNA copy numbers have been shown to have higher growth rates and exhibit copiotrophic lifestyles (Klappenbach et al., 2000; Stevenson and Schmidt, 2004; Yano et al., 2013; Roller et al., 2016). Marine sites during the 2018 hurricane season had higher average rRNA (*rrn*) copy number compared to estuarine sites during the 2018 hurricane season and all sites from non-hurricane conditions (Mann–Whitney U-test: *W* = 120, *p* < 0.001; Figure 5).

**Figure 6:**
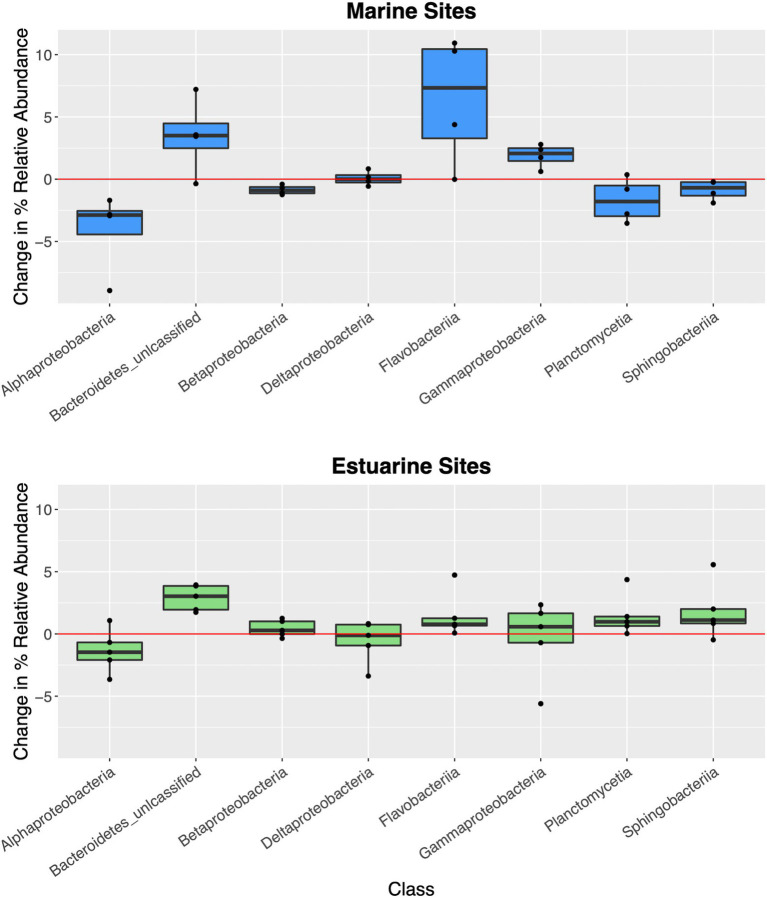
Boxplots of site-specific changes to microbial community composition in correlation with the 2018 hurricane season. Each data point represents difference in percent relative abundance of select taxonomic groups between sampling sites during the 2018 hurricane season and geographically similar sampling sites from 2019. Red line represents no change in percent relative abundance. Positive abundance values represent taxa that were more abundant during 2018 hurricane season, while negative abundance values represent taxa that were less abundant during 2018 hurricane season. Marine sites (>25 ppt, blue boxes) had higher proportion, or relative abundance of *Flavobacteriia*, *Gammaproteobacteria*, and unclassified *Bacteroidetes* while having lower relative abundance of *Alphaproteobacteria* in surface waters compared to non-hurricane conditions. Hurricane-influenced estuarine sites (<25 ppt, green boxes) were generally characterized by smaller changes in site-specific relative abundance, but nonetheless showed increased relative abundance of unclassified *Bacteroidetes* and decreased relative abundance of *Alphaproteobacteria* compared to non-hurricane conditions.

Investigating changes to microbial community composition on a site-specific basis and in correlation to hurricane season, marine sites (>25 ppt) had higher proportion, or relative abundance of *Flavobacteriia*, *Gammaproteobacteria*, and unclassified *Bacteroidetes* while having lower relative abundance of *Alphaproteobacteria* in surface waters compared to non-hurricane conditions (Figure 6; Supplementary Figure S3). Hurricane-influenced estuarine sites (<25 ppt) were generally characterized by smaller changes in site-specific relative abundance, but nonetheless showed increased relative abundance of unclassified *Bacteroidetes* and decreased relative abundance of *Alphaproteobacteria* compared to non-hurricane conditions. These trends were further confirmed with SIMPER analysis showing the most influential classes in determining community composition between hurricane and non-hurricane samples at marine sites (*Flavobacteriia*: 15.62%; *Gammaproteobacteria*: 10.88%; unclassified *Bacteroidetes*: 9.77%; *Planctomycetes*: 6.26%; *Alphaproteobacteria*: 6.21%; and *Sphingobacteria*: 4.45%) and at estuarine sites (*Gammaproteobacteria*: 9.97%; *Alphaproteobacteria*: 7.62%; unclassified *Bacteroidetes*: 7.19%; *Deltaprotebacteria*: 4.98%; and *Betaproteobacteria*: 4.46%). When comparing taxonomic differences between environment types, *Flavobacteriia*, *Alphaproteobacteria*, and unclassified *Bacteroidetes* classes were more prevalent in surface waters during hurricane conditions, while *Gammaproteobacteria* and *Deltaproteobacteria* were more prevalent in the sediment during hurricane conditions (Supplementary Figure S4). Taxonomic classes that were more shared across hurricane-influenced sediment and surface water samples included unclassified *Proteobacteria*, *Planctomycetia*, and *Sphingobacteriia*.

### Shifts in Indicator Taxa and Prevalent Metabolic Pathways in Hurricane-Influenced Coastal Systems

**Figure 7:**
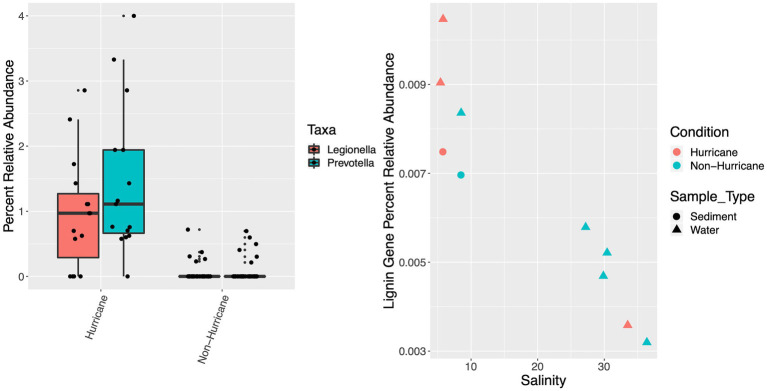
Boxplot of percent relative abundance, or proportion of potential pathogens *Legionella* and *Prevotella* among classified genus-level taxa (left), and scatterplot of percent relative abundance of potential lignin-degradation genes across metagenomic samples (right). Hurricane condition samples contained significantly higher relative abundance of *Legionella* and *Prevotella* (mean: 1.21%; SD: 1.03%) compared to non-hurricane conditions (mean: 0.05%; SD: 0.15%; Mann–Whitney U-test: *W* = 261.5, *p* < 0.001).

Collectively, both estuarine and marine hurricane-influenced sampling sites contained significantly higher proportion, or relative abundance of pathogenic indicator taxa (i.e., *Legionella* and *Prevotella*; mean: 1.21%; SD: 1.03%) among classified genus-level taxa compared to non-hurricane conditions (mean: 0.05%; SD: 0.15%; Mann–Whitney U-test: *W* = 261.5, *p* < 0.001; Figure 7). Hurricane-influenced sites also exhibited higher relative abundance of lignin-degradation genes (surface water and sediment combined; average: 7.64 × 10^−3^%; SD: 2.97 × 10^−3^%) compared to non-hurricane conditions (average: 5.90 × 10^−3^%; SD: 1.80 × 10^−3^%), although metagenomic sample numbers limit statistical significance (Figure 7). The 2018 hurricane season was further correlated with decreased presence of important functional gene pathways at marine sites (i.e., the glycolysis pathway: 77.7% complete; citrate cycle: 87.5% complete; glyoxylate cycle: 80% complete; reductive pentose phosphate cycle: 63.6% complete; and 3-hydroxypropionate bi-cycle: 0% complete) compared to marine sites during non-hurricane conditions on average (i.e., the glycolysis pathway: 97.2% complete; citrate cycle: 100% complete; glyoxylate cycle: 100% complete; reductive pentose phosphate cycle: 90.9% complete; and 3-hydroxypropionate bi-cycle: 87.5% complete; Supplementary Figure S5), although metagenomic sample numbers limit statistical significance. Hurricane-influenced sites (marine and estuarine) also showed an increased diversification in nitrogen cycling genes [i.e., increased presence of ammonia oxidation genes (*amoA*), nitrification genes (nitrite reductase), denitrification genes (nitrate reductase and *nirQ*), and nitrogen fixation genes (*nifU*)] within surface waters (Supplementary Figure S6).

Water quality measurements (i.e., TSS, DOC, and TDN in filtrates) did not vary between the 2018 hurricane season and non-hurricane season in this study (Supplementary Figure S7). Nonetheless, these variables did correlate with microbial community dynamics (Supplementary Figure S8). Communities were significantly correlated with salinity, TSS, TDN, and DOC (PERMANOVA test: salinity: *R*^2^ = 0.089, *p* < 0.001; total suspended solids: *R*^2^ = 0.045, *p* < 0.01; total dissolved nitrogen: *R*^2^ = 0.033, *p* < 0.05; and dissolved organic carbon: *R*^2^ = 0.362, *p* < 0.001).

## Discussion

### Contrasting Correlations Across Habitats, Domains, and Putative Cell Activity

Spanning from 2016 to 2019, a total of 28 surface water samples and 35 sediment samples were collected at sites in North Carolina within the Albemarle Sound, Pamlico Sound, Oregon Inlet, and along an E-W transect toward the edge of the continental shelf and Gulf Stream. Bacterial communities within surface waters were correlated with shifts in community composition during the 2018 hurricane season (with or without the inclusion of 2016 samples), potentially affecting ecosystem function within the area. These community shifts within surface waters persisted up to 77 days after passage of the first sequential storm (Hurricane Florence), suggesting that extreme storms likely affected biogeochemical processes such as carbon cycling at this study site for a large portion of the 2018 hurricane season. These extended periods of microbial community disturbance could be a result of the long residence times often observed in this region for nutrients within the water column, as well as the general hydrological characteristics of the region (Paerl et al., 2001). The transport of freshwater microbes seaward and their subsequent interactions with marine carbon sources and marine microbial populations could further compound the scope of disruption impacting the local ecosystem following extreme storm events. The extremely low-lying characteristics of the region probably also contribute to inland flooding and subsequent nutrient discharge following tropical storms and hurricanes (Rudolph et al., 2020). The high nutrient loads accompanying these inland wetlands and agricultural areas could potentially fuel microbial production in estuarine and marine environments downstream, contributing to the shifts in the bacterial community composition and functional potential observed in this study. The lack of correlation between sediment microbial communities and the 2018 hurricane season suggests that any potential settling of terrestrial microbes from the water column onto the sediment following the storm events are predicted to have not affected sediment microbial community structure or function within the sampling time frame of this study (i.e., hurricane signals within the sediment may have returned to baseline conditions prior to our sampling). Further, these sediment communities may also be more resistant or resilient to system-wide disturbances and geochemical fluctuations compared to those within the water column (Bowen et al., 2011). This would be consistent with sediment communities exhibiting high rates of cell dormancy, inactivity, and endosporulation (Luna et al., 2002; Shade et al., 2012; Wörmer et al., 2019).

Unlike bacterial communities, archaea within surface waters did not exhibit shifts in community composition during the 2018 hurricane season. One explanation for this could be that Archaea exhibit lower taxonomic diversity in terrestrial soil environments compared to other environment types (0.72 ± 0.71 Shannon index value in soils compared to 1.70 ± 0.63 in marine sediments; Auguet et al., 2010). This probably limits the relative abundance of archaeal cells that get flushed seaward due to river surging and flooding. Marine archaea are mostly comprised of nitrifiers (e.g., *Thaumarchaeota*, or ammonia-oxidizing archaea) and heterotrophs (e.g., *Euryarchaeota*; DeLong, 1992; Santoro et al., 2019). These ammonia oxidizers are often considered oligotrophs and thus may not exhibit significant shifts in abundance during hurricane season. Similarly, *Euryarchaeota* may also be unaffected due to their known roles in breakdown of phytoplankton produced organic matter (Orellana et al., 2019). The lack of correlation between archaea and hurricane season could alternatively be viewed as a form of resistance or resilience to these disturbance events compared to bacterial communities. The vast diversity of archaeal metabolism types such as methane and alkane oxidation and bacterial symbiosis have only recently started to come to light (Baker et al., 2020), and this metabolic diversity could potentially play a role in the stability of archaeal communities during hurricane conditions. There could also be methodological limitations to identifying archaeal shifts due to primer selections leading to sequencing biases. Further studies focusing on increased metagenomic sequencing depth and using a greater number of samples could help to identify trends in archaeal genes and genomes responding to pulse disturbances such as hurricanes.

The overall bacterial and archaeal responses to extreme storms in this study were further confirmed by RNA-based microbial profiling. The differences seen between DNA and RNA profiles across bacterial and archaeal domains suggest that not all microbial cells within the sediment were active (Figures 3B,D). The greater significant difference observed for putative cell activity (DNA vs. RNA) across sediment microbial communities rather than the influence of hurricane conditions supports the occurrence of cell dormancy, inactivity, and endosporulation expected within sediments (Luna et al., 2002; Wörmer et al., 2019). Meanwhile within surface waters, both total and active portions of bacterial community compositions were significantly correlated with hurricane conditions (i.e., differences in microbial community composition during hurricane conditions were not merely artifacts of inactive or dead microbial cells; Figure 3A). Although the active and inactive portions of the microbial communities were important determinants of functional potential during the 2018 hurricane season, the most significant effects were observed across domains (i.e., archaea vs. bacteria) and environment types (i.e., sediment vs. surface water). Results from the community composition analysis suggest that both archaeal and sediment communities may be more resilient or resistant to hurricane conditions compared to bacterial communities within surface waters. The potential resistance of sediment communities is not surprising given that sediment environments exhibit less environmental fluctuations compared to within the water column, but the suggested resistance of archaeal communities in the water column could indicate a community stabilizing effect provided by archaea during environmental disturbance scenarios. Future studies focusing on increased resilience and resistance of archaea compared to bacteria within coastal aquatic systems could build upon the results in this study.

### Shifts in Microbial Community Functional Potential During Hurricane Season

The whole-community trends above can be further strengthened by analysis of rRNA copy number, a proxy often used for inferring metabolic lifestyles within microbial communities. The increase in average rRNA gene copy number found at marine sites during the 2018 hurricane season in this study suggests increased abundance of copiotrophic microbial lifestyles (Figure 5), which supports the likelihood of increased offshore nutrient concentrations as a result of hurricane floodwater facilitated nutrient transport. These rRNA copy number results ultimately provide additional context to the whole-community disruption trends presented above. Further, increased nutrients and copiotrophs could potentially lead to competitive conditions that are unfavorable toward contrasting microbial lifestyles (i.e., oligotrophy; Schut et al., 1997), and as a result could disrupt the stability and overall function of the ecosystem in this region.

Shifts in abundance of individual taxonomic classes in correlation with the 2018 hurricane season in this study further suggest floodwater nutrient input into coastal waters (Figure 6). For example, *Flavobacteriia* and *Gammaproteobacteria* were higher in abundance at hurricane-influenced marine sites and are common marine heterotrophs that can break down a wide range of simple and complex carbohydrates (Edwards et al., 2010; Lawes et al., 2016; Liu et al., 2019). Decreases in relative abundance of the dominant marine *Alphaproteobacteria* taxa could be correlated to the oligotrophic lifestyles that characterize many of their members (Morris et al., 2002; Luo et al., 2013). Changes in the relative abundance of these taxa during the 2018 hurricane season is consistent with either transport of high nutrient loads offshore following the storm events, or taxa enrichment within nearshore environments prior to microbial transport to distances offshore. Taxonomic trends at estuarine sites in relation to the 2018 hurricane season showed minimal changes to relative taxonomic abundances. This could be indicative of estuarine sites returning to “baseline” conditions faster than marine sites (i.e., our sampling time periods may have missed the hurricane signature within the estuaries). Further comparisons of taxonomy across environment types, our results suggest that taxa more unique to sediment (*Gammaproteobacteria* and *Deltaproteobacteria*) may be stronger contributors to whole community resistance or resiliency compared to the shared taxa, while conversely, taxa more unique to surface waters (*Flavobacteriia*, *Alphaproteobacteria*, and unclassified *Bacteroidetes*) may be more vulnerable or less resistant or resilient compared to shared taxa during hurricane conditions (Supplementary Figure S4). Additional studies focusing on these taxonomic tendencies could provide more detail on functional impact of extreme storms and the potential resistance and resiliency toward environmental disturbance.

The increased relative abundance of pathogenic taxa represents another significant correlation with the 2018 hurricane season that has important implications for ecosystem health (Figure 7). This could result in an overall decrease in water quality and an increase in human health risks for coastal areas in North Carolina impacted by the 2018 hurricane season and potentially for future hurricane seasons. The exact source of these detected pathogens is not certain, but terrestrial runoff is the most likely scenario as both *Legionella* and *Prevotella* are well documented inhabitants of freshwater systems in the built environment and are often fecal indicators (Bennett and Bentham, 2006; Stewart et al., 2008; Arfken et al., 2015; Adyasari et al., 2019). The distribution of these specific pathogens could be unique to the Eastern North Carolina region as a result of the high concentrations of agricultural and animal production within this rural area (Niedermeyer et al., 2020), and further investigations following extreme storm events could be beneficial in supporting this regionally-specific trend.

There were no significant correlations between water quality variables and the 2018 hurricane season (Supplementary Figure S7) as noted earlier. This dissimilarity from the microbial results above suggests that microbes may have a higher residence time within the water column compared to hydrologic properties measured in this study (i.e., high nutrient signals may have returned to baseline prior to our sampling), or that water quality variables not quantified may have had an effect on the microbial community dynamics observed. Alternatively, another reasonable explanation for the variability could be the difference in size filtration between the dissolved geochemical variables (0.7 μm) and the microbial pool (0.2 μm). Microbial communities may have been more significantly influenced by submicron (<0.7 μm) sized organic materials, as colloidal and truly dissolved materials have significant differences in their nutrient and DOC associations (Guo et al., 1994; Herbert and Bertsch, 1995). Future studies aimed at capturing a wider spectrum of nutrient types and filtration sizes during hurricane season could potentially address the discrepancy between nutrients and microbes found in this study.

It is important to note that sampling for this study was conducted within different seasons of each year, and environmental factors associated with seasonality (e.g., length of day, temperature) could have therefore impacted microbial community dynamics. It is equally important to take the results themselves into context when determining potential degrees of influence from seasonality. For example, our results support the type of microbial response one would expect from large amounts of terrestrial organic material input into a marine system (i.e., decreased community evenness and diversity, and increased copiotrophic lifestyles and metabolic pathways; McGovern et al., 2020; Delpech et al., 2021; Figueroa et al., 2021). Previous studies have demonstrated correlation between seasonality variables (e.g., temperature) and cyclicity in microbial community compositions (Ward et al., 2017). Shorter lengths of daylight have also been correlated with increased diversity indices (Gilbert et al., 2012), and in the absence of storm derived floodwater influence, these findings are opposite of those in this study (Crump et al., 2009). Our results further argue against solely a seasonal trend, as neither sediment nor archaeal community compositions saw significant correlation with the hurricane season. For these reasons, we argue that important insight can still be gained from the results in this study even withstanding the potential for seasonal influences. We further suggest that for our geographic region of study, perhaps tropical storms and hurricanes are important to monitor alongside seasonality in order to predict changes in functional potential of microbes contributing to the health of an ecosystem.

### Correlations of Metagenomic Data With Hurricane Season

Investigations beyond taxonomy into functional gene distributions across hurricane conditions help to link taxonomy to function within these coastal ecosystems impacted by hurricanes. For example, lignin-degradation genes can be used as a proxy for tracking terrestrial material input to coastal marine systems. Lignin-degradation genes in this study were more abundant during the 2018 hurricane season (Figure 7), which is consistent with increased input of bulky, recalcitrant carbon sources such as lignin into coastal waterways during the 2018 hurricane season (Yan et al., 2020). Note that lignin-degradation gene abundance was also higher at lower salinity sites as expected from increased proximity to these terrestrial sources. Lignin-based carbon sources are important to investigate because they could potentially persist in marine systems for extended time periods due to their recalcitrant nature and the complex degradation process required for their removal (Crawford and Crawford, 1980; Brown and Chang, 2014; Kamimura et al., 2019). The presence of these lignin-degradation genes supports the likelihood of recalcitrant terrestrial carbon sources persisting in marine systems during hurricane conditions, as well as an increase in carbon remineralization potential due to terrestrial microbes and organic matter introductions to the marine system.

The disruptions to microbial community composition and functional potential observed in this study are further supported by important shifts in metabolic pathways during hurricane conditions compared to non-hurricane conditions (Supplementary Figures S5, S6). Decreased presence of pathways involved in the breakdown of simple sugars and carbohydrates *via* the glycolysis pathway and citrate cycle could indicate a shift toward increased breakdown of more complex biopolymers such as lignin, or a general shift toward alternative biosynthesis pathways. This persistence could be one avenue leading to microbial community disruptions without a significant difference observed for water quality measurements taken during this study. The decreased presence of genes associated with the glyoxylate cycle at hurricane-influenced marine sites is notable because this pathway is utilized for mineralization of organic matter *via* acetate as an intermediate carbon source and has been positively correlated with SAR11 relative abundance, the most abundant marine heterotroph (Zhuang et al., 2019). The lower relative abundance of this gene pathway at marine sites could be a result of injections of terrestrial microbes into the system causing an imbalance within the microbial community, or from shifts toward communities dominated by copiotrophs (i.e., within the *Flavobacteriia* and *Gammaproteobacteria* clades) rather than oligotrophs such as SAR11 (*Alphaproteobacteria*). Additionally, marine sites during hurricane season recovered fewer genes for reductive pentose phosphate cycle, an important pathway for photosynthesis (Bassham, 1979), and for 3-hydroxypropionate bi-cycle, a carbon dioxide fixation pathway for photosynthetic bacteria (Tabita, 2009; Zarzycki and Fuchs, 2011). This could potentially suggest less primary production occurring at hurricane-influenced marine sites compared to non-hurricane conditions and would be consistent with an overall shift at offshore sites from autotroph-dominated to heterotroph-dominated metabolisms. The increased presence of ammonia oxidation genes (*amoA*), nitrification genes (nitrite reductase), denitrification genes (nitrate reductase and *nirQ*), and nitrogen fixation genes (*nifU*) are consistent with an overall increase in nitrogen-based runoff to the coastal marine system during hurricane conditions, likely due to riverine and wetland flooding. While the metabolic pathway data from metagenomic sequencing represents functional potential only, future studies on hurricane-influenced metabolic pathways could benefit from RNA-based transcriptomics analyses to better understand active functional roles of these pathways.

### Conclusion

Overall, this study highlights the impact of extreme storms on coastal microbial communities and suggests that these impacts are highly dependent on the geographic characteristics of the study site. Other studies have shown variable degrees of impact from extreme storms on microbial communities, and our results provide evidence implying significant disruption to coastal ecosystem function throughout the latter part of the 2018 hurricane season (October through November). As opposed to a discrete, stochastic response to a single disturbance, the continual nature of hurricane season in Eastern North Carolina suggests a corresponding longevity for microbial impacts and disruptions to biogeochemical cycles. Of particular importance, we found contrasting correlations among habitats (i.e., surface water vs. sediment), taxonomic domains (i.e., bacteria vs. archaea), and putative cell activity (i.e., DNA vs. RNA). Moreover, trends in geochemical variables (i.e., TSS, DOC, TDN) were uncoupled from microbial populations isolated in this study. The weak correlations evident between microbial community composition and water chemistry (salinity excluded) imply that microbes were either more sensitive to extreme storm disturbances or were affected for a longer period of time compared to hydrologic properties measured in this study. These results further support the context-dependent nature of pulse disturbance scenarios, and understanding these variable contexts is critical for improving the link between taxonomy and function of hurricane-influenced microbial communities. These communities were further characterized by decreases in taxonomic evenness and Shannon diversity, as well as shifts toward individual taxa and metabolic pathways characteristic of increased nutrient concentrations and decreased water quality during hurricane conditions. The shifts in relative abundance of copiotrophs, pathogenic taxa, and carbon and nitrogen cycling genes suggest a potential transition from autotroph-dominated to heterotroph-dominated microbial communities during hurricane conditions. Further studies across a broader range of water quality variables and size filtrations could potentially build upon the disconnect between microbes and geochemistry observed in this study.

The lasting impacts on microbial communities from hurricanes may cause significant disruptions to nutrient cycling within a geographic area that fuels a large portion of primary production in the North Atlantic Ocean (i.e., The Gulf Stream). Significantly altered marine microbial communities due to extreme storms would have important consequences for regional nutrient cycling with increased atmospheric CO_2_ inputs being counter-productive for warming global climates. It is important to note that the processes shaping microbial community function are highly complex and the correlations with the 2018 hurricane season shown in this study may also be influenced by other unknown external processes. Sampling across additional years was outside the scope of this project, but the changes in microbial community composition and functional potential observed during the 2018 hurricane season can ideally be used as building blocks for future studies aimed at identifying annual trends, resolving seasonality, and predicting the microbiological impact of increased intensities and frequencies of extreme storms that are thought to accompany a warming global climate. As we expand our understanding of microbial responses to extreme weather disturbances, we can further improve our knowledge of the potential fate of future global oceans.

## Data Availability Statement

The datasets presented in this study can be found in online repositories. The names of the repository/repositories and accession number(s) can be found at: https://www.ncbi.nlm.nih.gov/, PRJNA779973.

## Author Contributions

CG was responsible for project preparation, investigation, interpretation of results, and writing of the original draft. SR contributed to project preparation, investigation, and manuscript revisions. SM was responsible for funding acquisition, project administration, project investigation, and manuscript revisions. DC contributed to project investigation and manuscript revisions. EF was responsible for project conceptualization, preparation, investigation, interpretation, and manuscript revisions. All authors contributed to the article and approved the submitted version.

## Funding

This work was supported by National Science Foundation Division of Ocean Sciences Chemical Oceanography Award #1902496.

## Conflict of Interest

The authors declare that the research was conducted in the absence of any commercial or financial relationships that could be construed as a potential conflict of interest.

## Publisher’s Note

All claims expressed in this article are solely those of the authors and do not necessarily represent those of their affiliated organizations, or those of the publisher, the editors and the reviewers. Any product that may be evaluated in this article, or claim that may be made by its manufacturer, is not guaranteed or endorsed by the publisher.

## Footnotes

^1^Available at: http://rdp.cme.msu.edu/classifier/classifier.jsp

^2^Available at: https://www.biorender.com
