# Supplementary material for: Coastal Microbial Communities Disrupted During the 2018 Hurricane Season in Outer Banks, North Carolina
Source: Front Microbiol. 2022 Jun 9;13:816573. doi: 10.3389/fmicb.2022.816573 (PMC9218724; doi:10.3389/fmicb.2022.816573)
Supplement: Supplementary file 1 [file Data_Sheet_1.docx]

Supplementary Material:

Table S1: General geophysical characteristics and sample types collected at each site

| **Site** | **Sampling Date** | **Latitude** | **Longitude** | **Salinity (ppt)** | **Water Depth (m)** | **Temp (°C)** | **Surface Water DNA Analysis** | **Surface Water RNA Analysis** | **Sediment DNA Analysis** | **Sediment RNA Analysis** |
| --- | --- | --- | --- | --- | --- | --- | --- | --- | --- | --- |
| 1 | 10/25/18 | 35.3766 | -75.8046 | 21.3 | 5.9 | N/A | Y | Y | Y | Y |
| 2 | 10/25/18 | 35.4877 | -75.6641 | 16.9 | 5.6 | 17.9 | Y | Y | Y | Y |
| 3 | 10/25/18 | 35.6484 | -75.6230 | 11.7 | 4 | 16.1 | Y | Y | Y | Y |
| 4 | 10/26/18 | 35.7030 | -75.6575 | 11.8 | 4.1 | 15.4 | Y | Y | Y | Y |
| 5 | 10/26/18 | 35.7606 | -75.5875 | 19.8 | 3.4 | 15.6 | Y | Y | Y | Y |
| 6 | 10/26/18 | 35.7710 | -75.5380 | 29.9 | 3.8 | 18.3 | Y | Y | Y | N |
| 7 | 10/26/18 | 35.7536 | -75.5275 | 29.3 | 1.4 | 19 | Y | Y | Y | N |
| 8 | 10/26/18 | 35.7949 | -75.6221 | 10.7 | 2.1 | 15.7 | Y | Y | Y | Y |
| 9 | 10/26/18 | 35.8782 | -75.7144 | 5.8 | 2.4 | 15.4 | Y | Y | Y | Y |
| 50 | 11/30/18 | 35.8065 | -75.5879 | 4.2 | 1.9 | 7.5 | Y | Y | Y | Y |
| 51 | 11/30/18 | 35.7817 | -75.5284 | 5.4 | 4.8 | 7.4 | Y | Y | Y | N |
| 52 | 11/30/18 | 35.8794 | -74.8322 | 33.5 | 35.3 | 14.6 | Y | Y | N | N |
| 53 | 11/30/18 | 35.9033 | -75.0908 | 32.4 | 32 | 13.9 | Y | Y | N | N |
| 54 | 11/30/18 | 35.8994 | -75.1953 | 31.4 | 37.8 | 13.6 | Y | Y | N | N |
| 55 | 11/30/18 | 35.8779 | -75.3627 | 30.2 | 25 | 13.2 | Y | Y | N | N |
| 100 | 7/2/19 | 35.8781 | -74.8375 | 29.9 | 356 | 25.4 | Y | N | N | N |
| 101 | 7/2/19 | 35.8802 | -75.0141 | 29.7 | 44.2 | 25.7 | Y | N | N | N |
| 102 | 7/2/19 | 35.8684 | -75.1331 | 29.8 | 30.5 | 25.8 | Y | N | Y | Y |
| 103 | 7/2/19 | 35.8066 | -75.4487 | 26.7 | 12 | 24.7 | Y | N | Y | Y |
| 104 | 7/2/19 | 35.7976 | -75.5171 | 27.2 | 11.4 | 24.3 | Y | N | Y | Y |
| 105 | 7/3/19 | 35.4560 | -75.7683 | 20.1 | 5.9 | 28.4 | Y | N | Y | Y |
| 106 | 7/3/19 | 35.4856 | -75.6656 | 18.7 | 5.6 | 28.4 | Y | Y | Y | Y |
| 107 | 7/3/19 | 35.6440 | -75.6269 | 15.9 | 4 | 28.7 | Y | Y | Y | N |
| 108 | 7/3/19 | 35.7596 | -75.5910 | 14.7 | 2.7 | 28.1 | Y | Y | Y | N |
| 109 | 7/3/19 | 35.7636 | -75.5307 | 14 | 1.3 | N/A | Y | Y | Y | N |
| 110 | 7/3/19 | 35.8591 | -75.6852 | 8.5 | 3 | 28.7 | Y | Y | Y | Y |
| ML1 | 3/17/16 | 35.0498 | -75.2631 | 36.4 | 98 | 20 | Y | Y | Y | Y |
| MM2 | 3/18/16 | 35.1378 | -75.1010 | 36.4 | 230 | 21 | N | N | Y | Y |
| XS1 | 3/22/16 | 35.7696 | -75.4736 | 30.4 | 20 | 9.3 | Y | Y | Y | Y |
| DD11 | 3/22/16 | 35.5609 | -74.8669 | 34.2 | 55 | 10 | N | N | Y | Y |


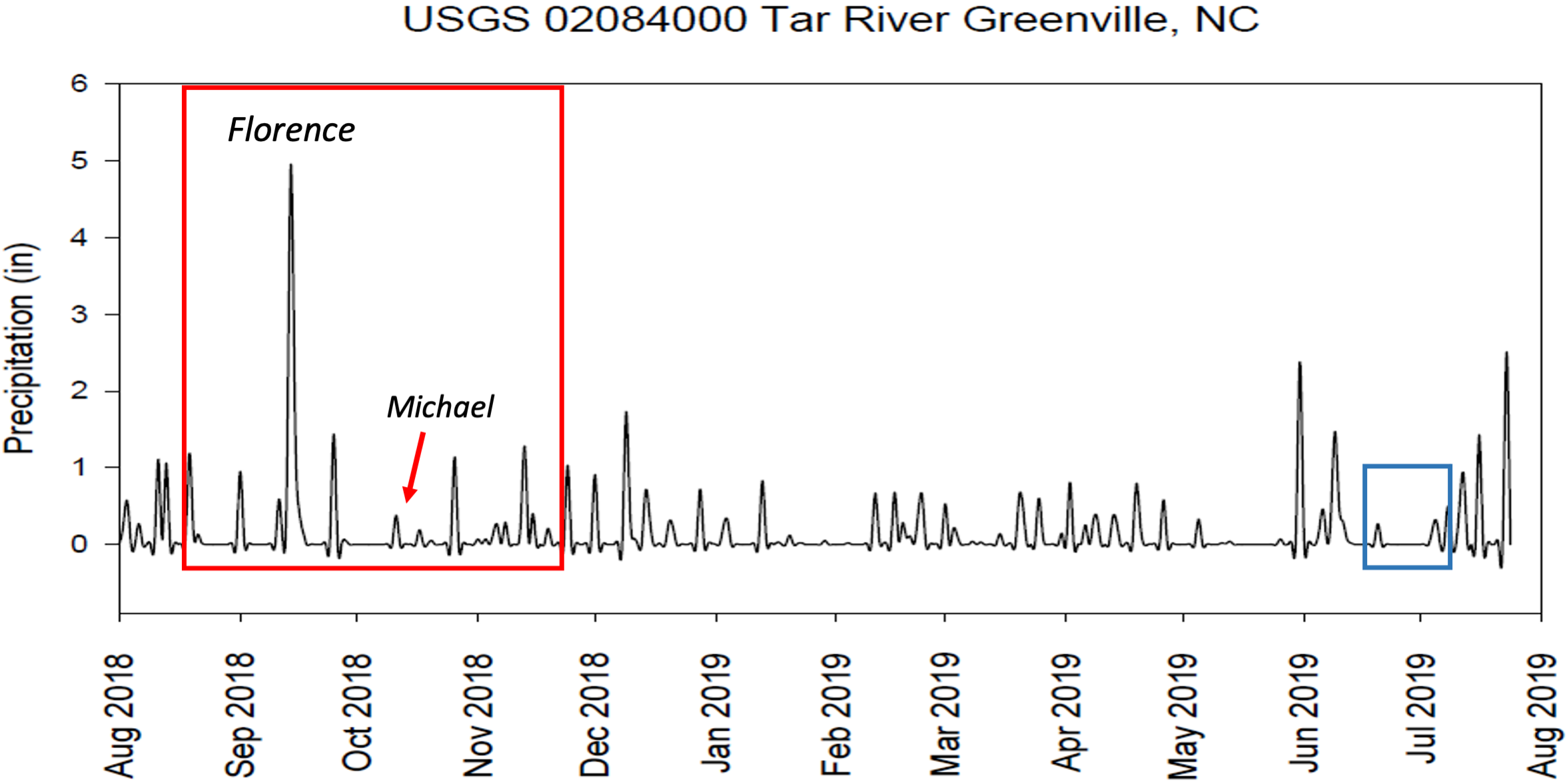


Fig. S1: Precipitation time-series from USGS monitoring station in Greenville, NC spanning 2018 and 2019 sample collections. 2018 hurricane season highlighted in red, and non-hurricane season from 2019 sampling period highlighted in blue.


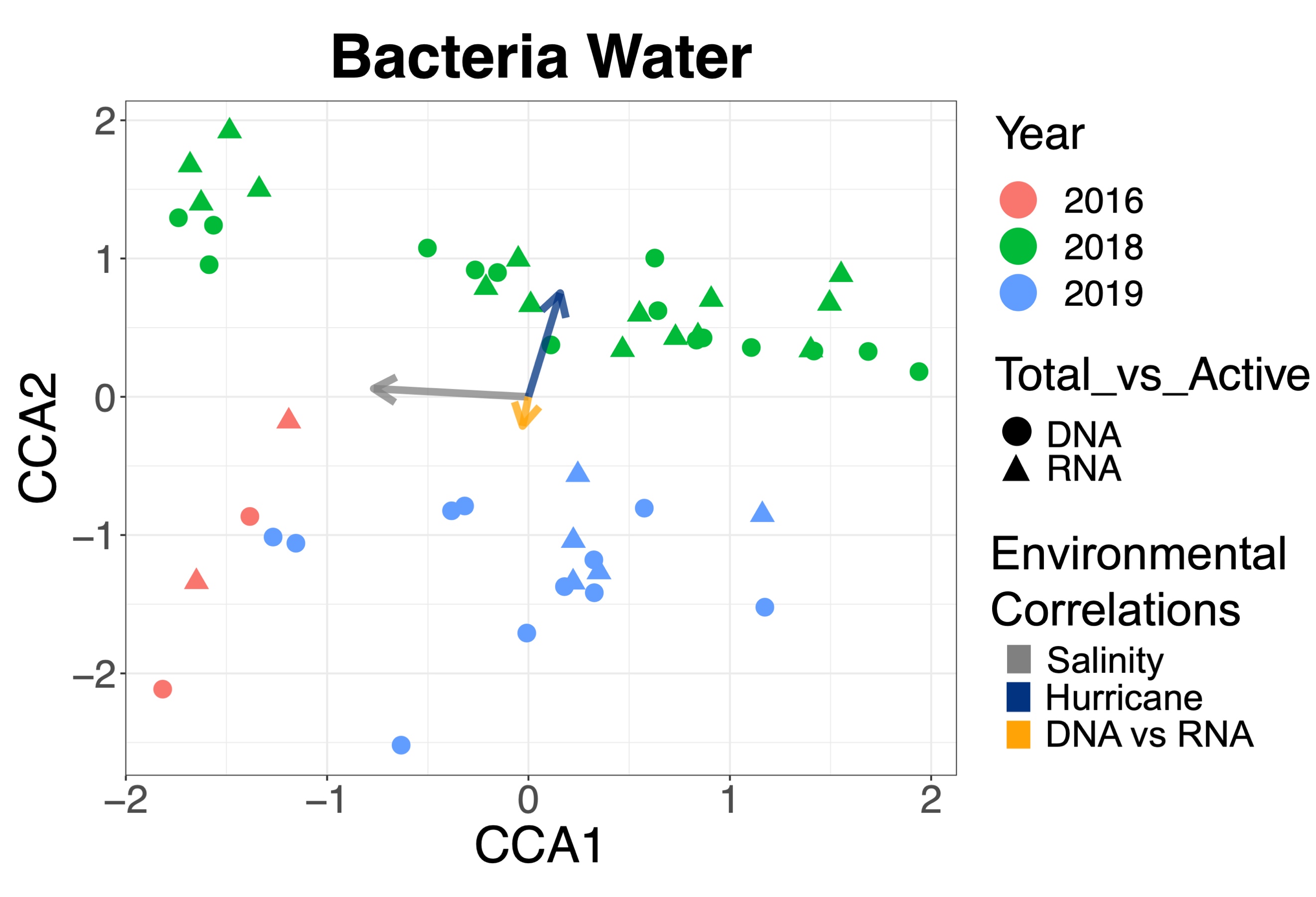
Fig. S2: Canonical Correspondence Analysis plot showing similarities in bacterial community composition across surface water samples. Each data point is one sample, and distance between points represent degree of similarity in community composition. Arrows represent influence from environmental variables, and length of arrow represents strength of correlation. Bacterial communities in non-hurricane condition samples from 2016 and 2019 were more similar to each other than to those taken during the 2018 hurricane season, although the limited number of samples from 2016 limit statistical significance.


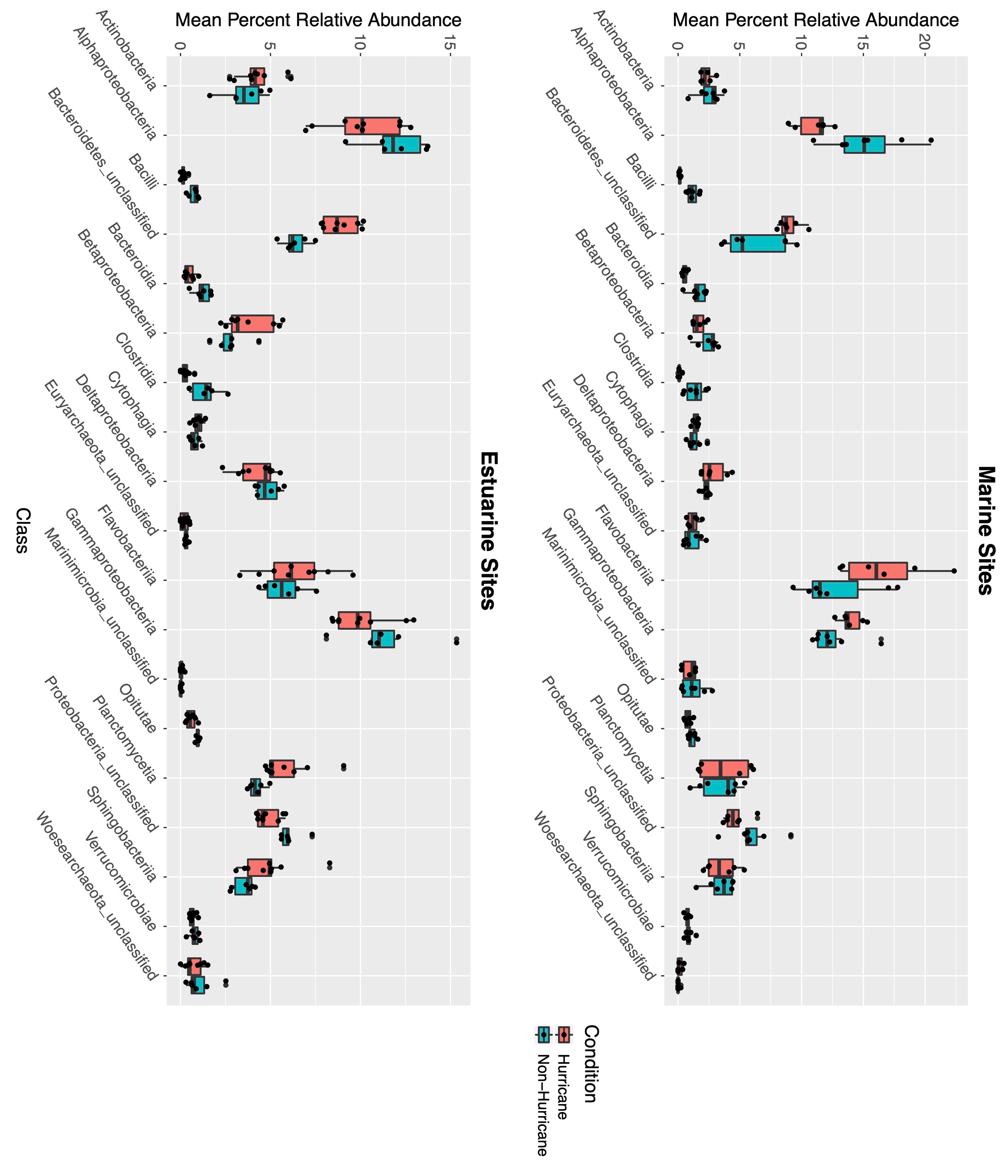


Fig. S3: Boxplots of average percent relative abundance of top microbial taxonomic classes (i.e., taxa that have the highest relative abundance across all samples). Individual data points represent individual samples. Marine sites during hurricane season had higher relative abundance of *Flavobacteriia*, *Gammaproteobacteria*, and unclassified *Bacteroidetes* in surface waters, while having lower relative abundance of *Alphaproteobacteria* and unclassified *Proteobacteria* compared to non-hurricane season. Estuarine sites (<25 ppt) during hurricane season were characterized by increased relative abundance of *Planctomycetia*, *Sphingobacteriia*, *Betaprotebacteria*, and unclassified *Bacteroidetes*, while having decreased relative abundance of *Gammaproteobacteria* and unclassified *Proteobacteria* compared to non-hurricane season.


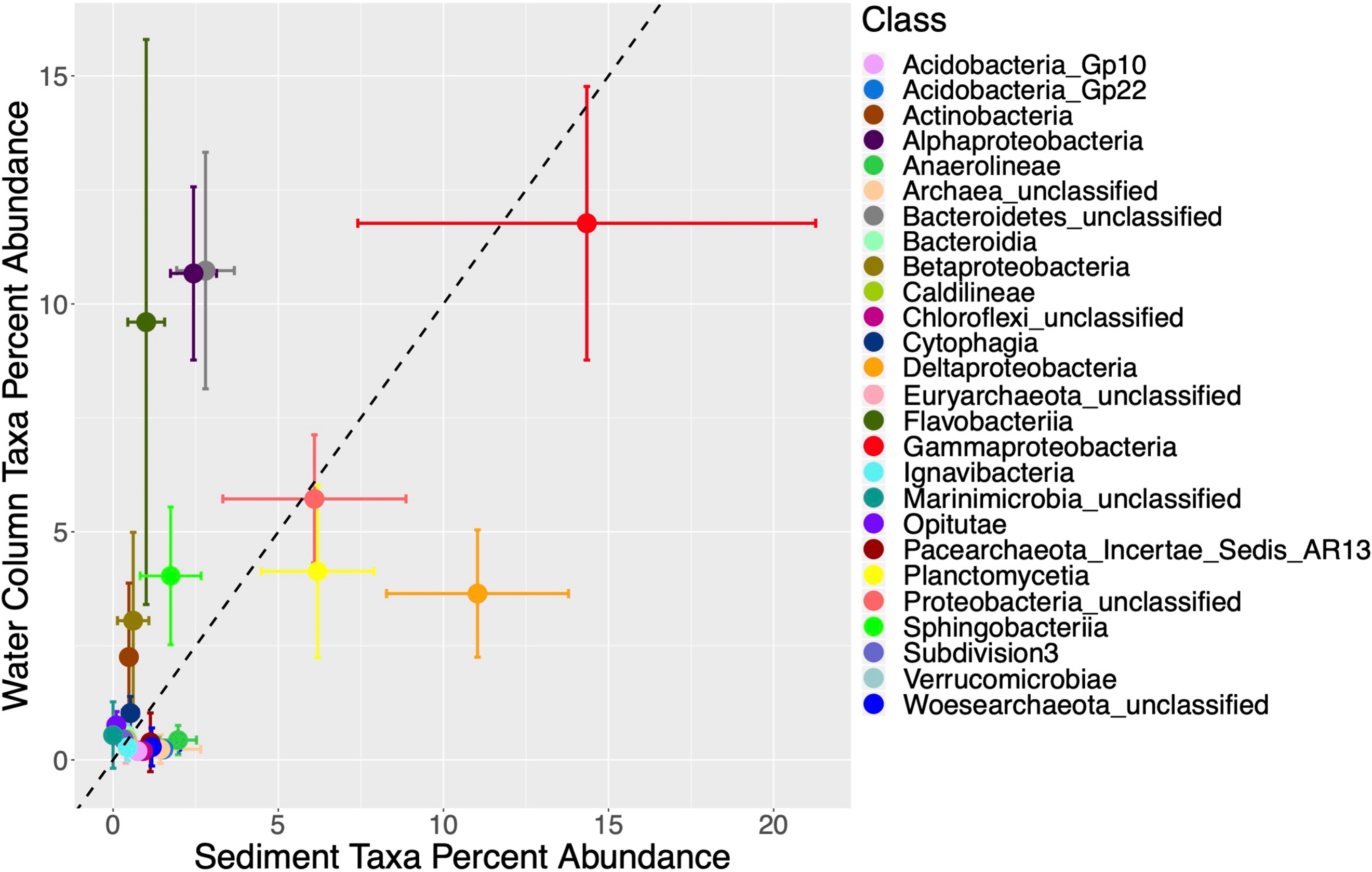


Fig. S4: Sediment taxa abundance plotted against water column taxa abundance at the class level during hurricane season. *Flavobacteriia*, *Alphaproteobacteria*, and unclassified *Bacteroidetes* were more prevalent in the water column while *Gammaproteobacteria* and *Deltaproteobacteria* were more prevalent in the sediment. Taxonomic classes that were more shared across sediment and water column samples (closest to the 1:1 ratio line) during hurricane season included unclassified *Proteobacteria*, *Planctomycetia*, and *Sphingobacteriia*. Data points represent average relative abundance and error bars represent standard deviation across samples.


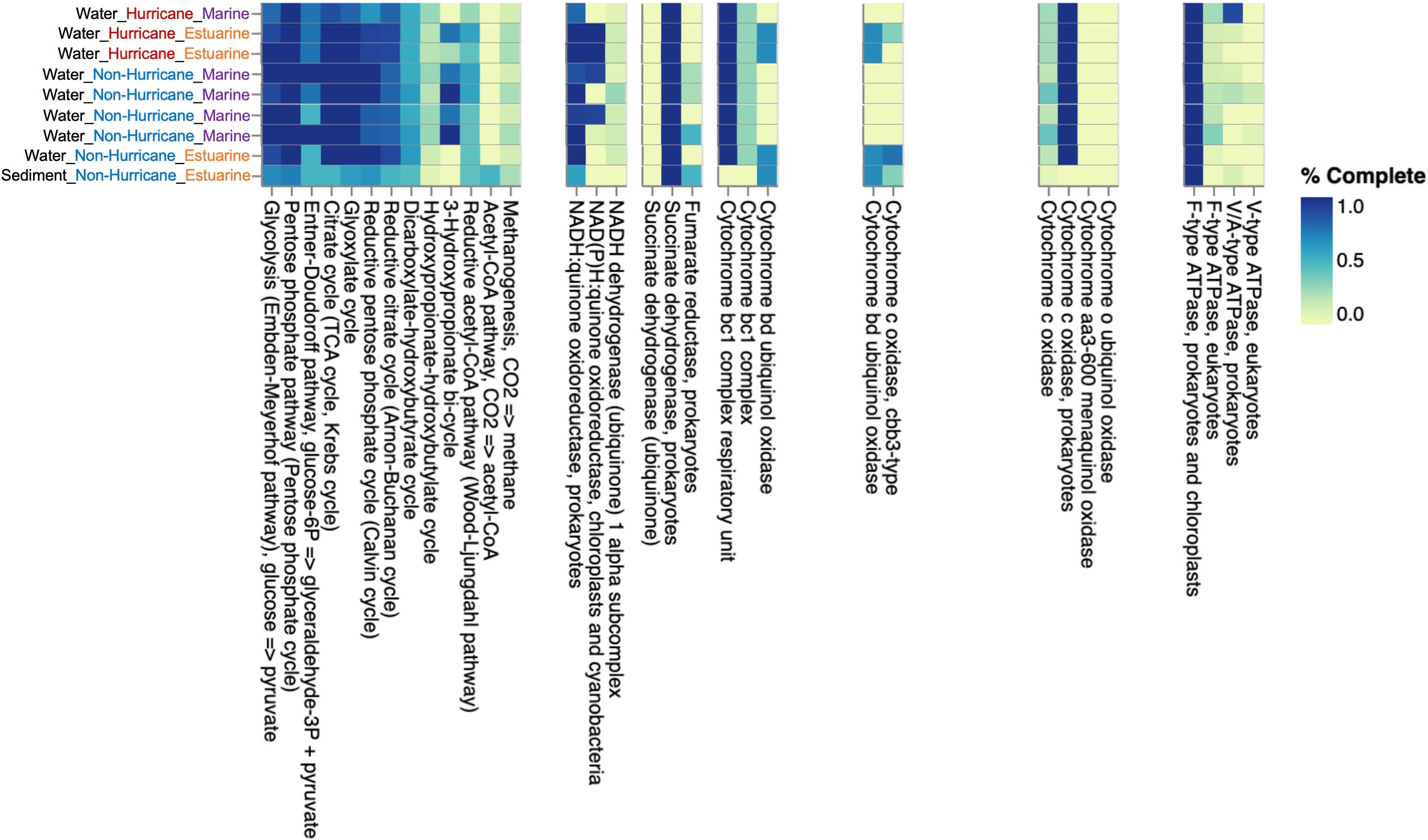


Fig. S5: Heatmap indicating percent completeness of metabolic pathways across samples sequenced with shotgun metagenomic methods. Marine sites during hurricane season had decreased relative abundance of genes involved in the glycolysis pathway, citrate cycle, glyoxylate cycle, reductive pentose phosphate cycle, and 3-hydroxypropionate bi-cycle pathways.


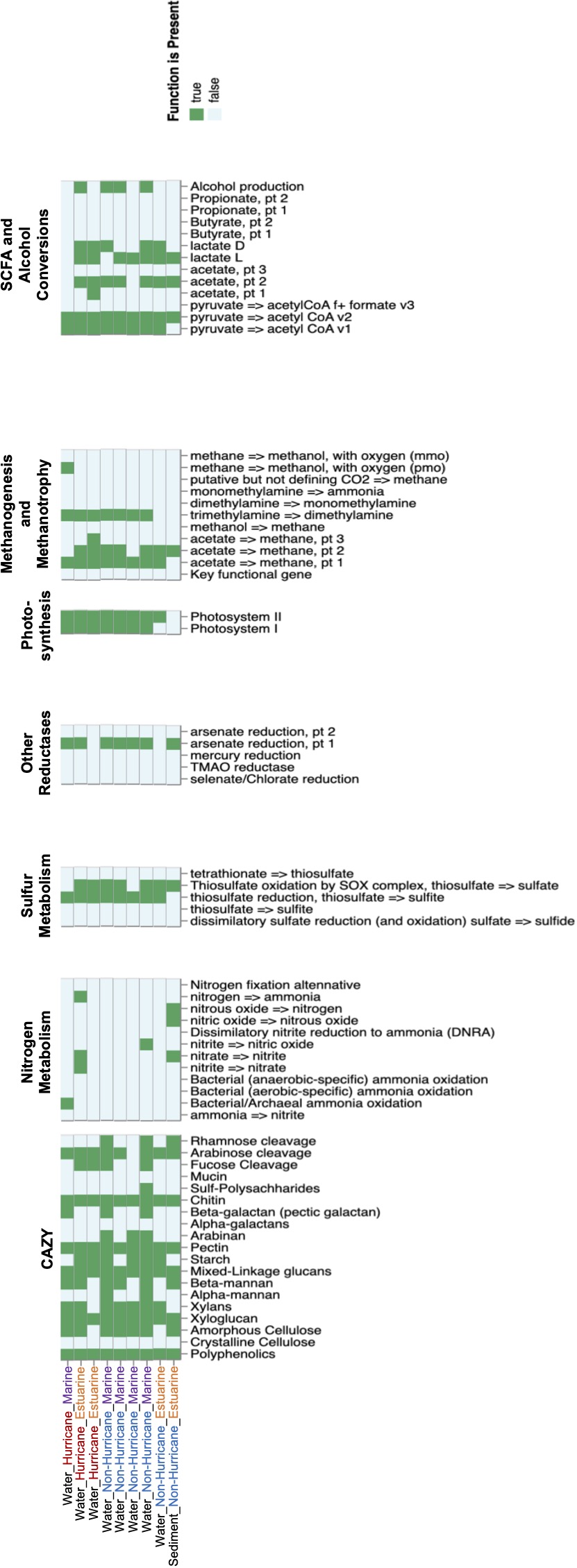


Fig. S6: Heatmap indicating presence or absence of metabolic pathways across samples sequenced with shotgun metagenomic methods. Marine and estuarine sites during hurricane season showed an increased diversity of nitrogen cycling pathways, including genes for ammonia oxidation, nitrification, denitrification, and nitrogen fixation pathways.


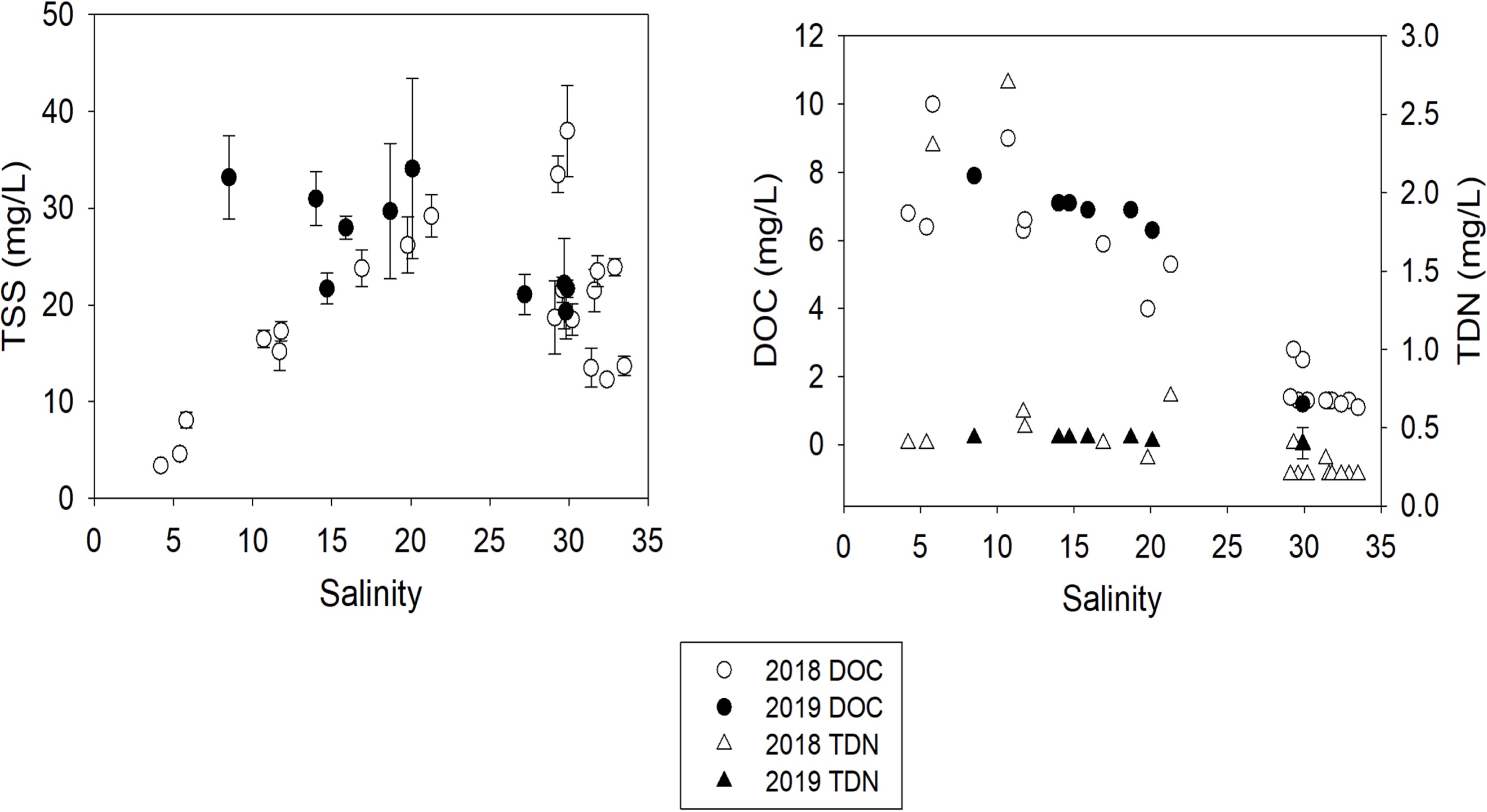


Fig. S7: Total suspended solids (TSS) against salinity (left); dissolved organic carbon (DOC) and total dissolved nitrogen (TDN) against salinity (right). Open symbols (left) represent samples collected during the 2018 hurricane season and solid symbols represent samples collected during non-hurricane season in 2019. There were no significant correlations between hurricane season and TSS, TDN, or DOC.


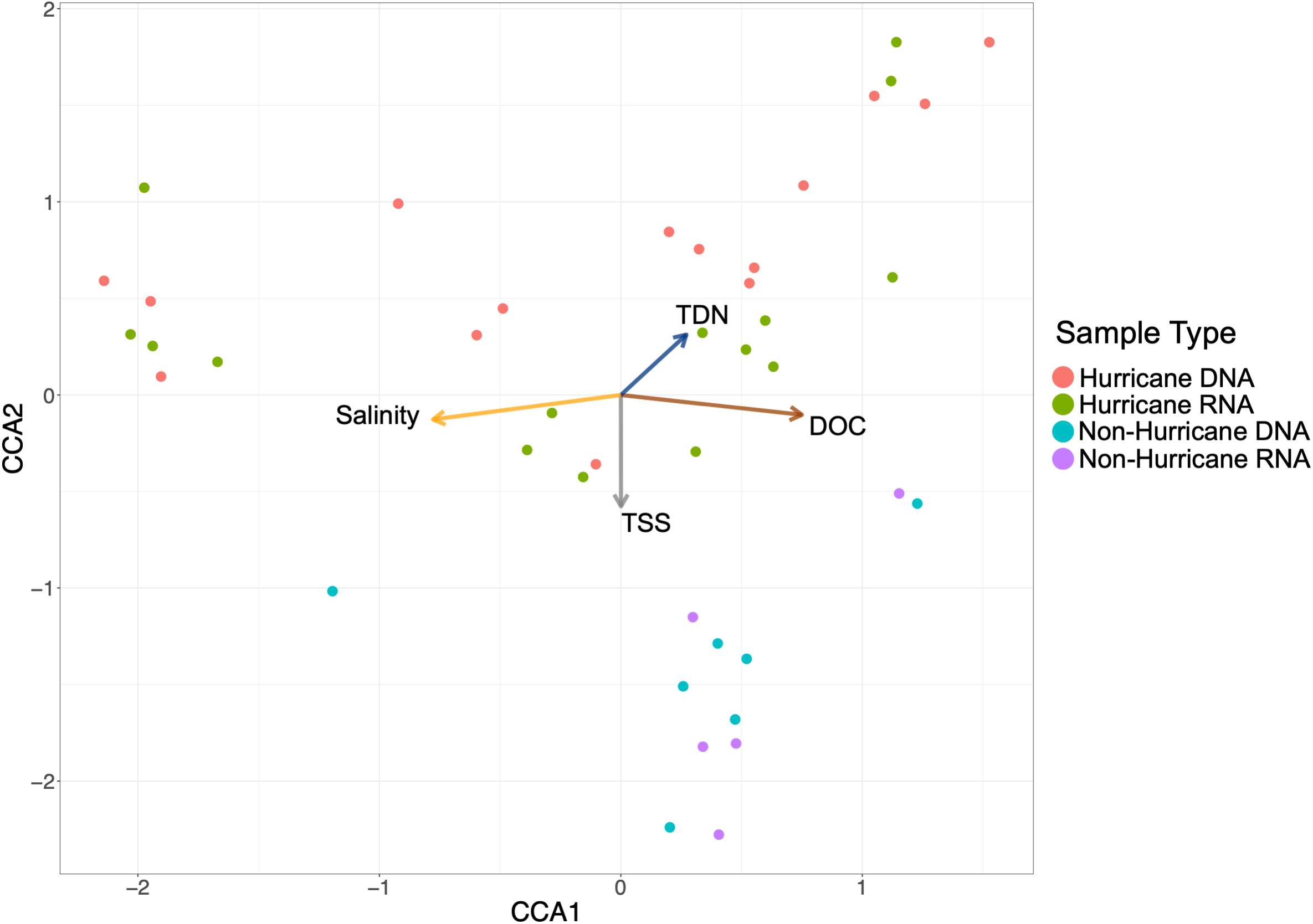


Fig. S8: Canonical Correspondence Analysis plot showing microbial community compositions in water column samples with influence of additional water quality variables. Communities were significantly correlated with salinity, total suspended solids (TSS), total dissolved nitrogen (TDN), and dissolved organic carbon (DOC). (PERMANOVA test: Salinity: R^2^ = 0.089, p < 0.001; Total suspended solids: R^2^ = 0.045, p < 0.01; Total dissolved nitrogen: R^2^ = 0.033, p < 0.05; Dissolved organic carbon: R^2^ = 0.362, p < 0.001).
